# A Novel LiDAR Echo Signal Denoising Method Based on the VMD-CPO-IWT Algorithm

**DOI:** 10.3390/s25206330

**Published:** 2025-10-14

**Authors:** Jipeng Zha, Xiangjin Zhang, Tuan Hua, Na Sheng, Yang Kang, Can Li

**Affiliations:** 1 School of Mechanical Engineering, Nanjing University of Science and Technology, Nanjing 210094, China; zhajipeng@njust.edu.cn; 2 Jincheng Nanjing Electromechanical Hydraulic Engineering Research Center, Aviation Industry Corporation of China, Nanjing 211106, China; syassht@163.com; 3 National Key Laboratory of Transient Physics, Nanjing University of Science and Technology, Nanjing 210094, China; snbox@njust.edu.cn (N.S.); ykang@njust.edu.cn (Y.K.); lican@njust.edu.cn (C.L.)

**Keywords:** LiDAR echo signal denoising, variational mode decomposition, Wasserstein distance, improved wavelet thresholding, Crested Porcupine Optimizer

## Abstract

Due to the susceptibility of LiDAR echo signals to various noise interferences, which severely affect their detection quality and accuracy, this paper proposes a joint denoising method combining Variational Mode Decomposition (VMD), Crested Porcupine Optimizer (CPO), and Improved Wavelet Thresholding (IWT), named VMD-CPO-IWT. The parameter-adaptive CPO optimization algorithm is employed to optimize the key parameters of VMD (decomposition level k, quadratic penalty factor α), effectively solving the challenge of determining the optimal parameter combination in the VMD algorithm. Based on the probability density function (PDF), the Wasserstein distance is used as a similarity metric to screen intrinsic mode functions. Subsequently, the IWT is applied to obtain the optimal wavelet threshold, which compensates for the shortcomings of traditional threshold methods while further suppressing both low-frequency and high-frequency noise in the signal, ultimately yielding the denoising result. Experimental results demonstrate that for both simulated signals and actual LiDAR echo signals, the VMD-CPO-IWT method outperforms Neighcoeff-db4 wavelet denoising (WT-db4), EMD combined with detrended fluctuation analysis denoising (EMD-DFA), and VMD combined with Whale Optimization Algorithm (VMD-WOA) in terms of improving the Signal-to-Noise Ratio (*SNR*) and reducing the Root Mean Square Error (*RMSE*). For the actual LiDAR echo signal at a detection range of 25 m, the *SNR* is improved by 13.64 dB, and the *RMSE* is reduced by 62.6%. This method provides an efficient and practical solution for denoising LiDAR echo signals.

## 1. Introduction

LiDAR is an integration of conventional radar technology and modern laser techniques, representing a powerful active remote sensing methodology. It offers distinct advantages, including high resolution, extensive detection range, and robust immunity to electromagnetic interference [1,2]. This technology plays a pivotal role in diverse applications such as atmospheric monitoring [3,4], target recognition [5,6], and 3D imaging [7]. However, LiDAR echo signals are highly susceptible to solar background light, detector dark current, and amplifier thermal noise, making the weak echo signals even more difficult to recognize [8,9,10]. The electronic thermal noise inherent in the system hardware is exacerbated under long operation or high temperature conditions, further affecting the detection accuracy [11]. Moreover, at long distances and in environments with low *SNR*, the environmental interference is enhanced and the echo power is reduced, resulting in echo signals that are highly susceptible to being annihilated in the noise. Therefore, the development of advanced noise reduction algorithms is crucial to improve the data quality and accuracy of LiDAR detection.

In recent years, several filtering algorithms applicable to LiDAR echo signal denoising have been extensively investigated, including the Fourier transform (FT), wavelet transform (WT), empirical mode decomposition (EMD), and VMD. These methods have demonstrated effectiveness under various operational scenarios. Among conventional signal processing approaches, FT extracts target signals by analyzing frequency distributions of noise and useful components. However, its inability to perform localized time-frequency analysis restricts its applicability to linear and stationary signals [12]. For LiDAR signal exhibiting nonlinear and non-stationary characteristics, FT often yields unsatisfactory denoising performance with significant signal distortion. Wavelet transform decomposes noisy signals into weighted sums of wavelets at different scales, employing predetermined thresholds to filter noise-contaminated coefficients. While WT offers excellent time-frequency localization, its performance is constrained by wavelet basis selection limitations and poor adaptability [13]. As an emerging denoising technique, EMD has gained widespread adoption in biomedical engineering, mechanical fault diagnosis, and audio processing [14]. This method adaptively decomposes signals into band-limited intrinsic mode functions (BLIMFs), making it suitable for non-stationary and nonlinear signals. Nevertheless, EMD suffers from critical drawbacks, including mode mixing and end effects during decomposition, which adversely impact denoising outcomes. VMD, introduced by Dragomiretskiy and Zosso in 2014, represents a novel adaptive signal decomposition approach fundamentally distinct from EMD [15]. VMD exhibits superior capabilities in processing nonlinear and non-stationary signals. Theoretically, whereas EMD employs recursive time-domain decomposition, VMD implements fully non-recursive frequency-based decomposition. Consequently, VMD not only overcomes mode-mixing issues inherent in EMD but also achieves enhanced filtering performance through its inherent Wiener filtering characteristics. VMD has been successfully implemented across multiple domains, including mechanical fault diagnosis, biomedical signal processing, and LiDAR signal enhancement [16,17,18].

However, to fully leverage the advantages of VMD, two critical parameters must be determined: the number of decomposition modes K and the quadratic penalty term α. These parameters are typically selected within specified ranges. In previous studies, they were often determined empirically based on convenience [19,20,21], substantially constraining VMD’s performance and potentially leading to inaccurate decomposition results. Several researchers have investigated methodologies for optimal parameter selection. Long et al. [22] employed Particle Swarm Optimization (PSO) to identify optimal VMD parameters. Although PSO can yield suitable parameters, it exhibits tendencies toward local optima trapping and relatively slow convergence. Li et al. [23] utilized the Whale Optimization Algorithm (WOA) to obtain optimal combinations of K and α. However, WOA’s performance is contingent upon initial population selection; inadequate coverage of the parameter space may prevent convergence to global optima. Gu et al. [24] refined the adaptive mode selection criterion for VMD to determine decomposition levels. Nevertheless, this approach requires manual threshold calibration based on specific scenarios, introducing operational complexity and subjectivity.

Beyond parameter optimization, the essence of VMD-based signal filtering lies in selecting relevant modes for signal reconstruction after decomposition. When applying VMD to noisy data, it is imperative to discriminate between the physical nature of each mode—whether it constitutes pure signal, pure noise, or hybrid components [25]. Zhou et al. [26] employed correlation-based Euclidean distance to identify pertinent modes for signal reconstruction. However, for signals exhibiting complex dynamic characteristics, this metric fails to comprehensively characterize inter-modal relationships, consequently impairing mode identification efficacy. Zhang, Y. et al. [27] adopted detrended fluctuation analysis (DFA) to establish thresholds distinguishing noise-dominant and signal-dominant BLIMFs. While DFA effectively measures long-range dependencies in non-stationary time series, it neglects correlations with simulated signals and lacks adaptability. Xinru Cao et al. [28] implemented a correlation coefficient thresholding method for selectively filtering noise-contaminated BLIMFs. Nevertheless, threshold determination in this approach relies heavily on empirical trial-and-error, introducing substantial subjectivity in practical applications.

Aiming at the shortcomings of existing research, a denoising algorithm for pulsed laser echo signals based on parameter-optimized VMD is proposed. Firstly, the CPO is introduced to iteratively optimize the decomposition parameters of the classical VMD method, overcoming the limitation that traditional VMD cannot adaptively initialize parameters. Secondly, the Wasserstein distance is adopted as a criterion to discriminate between relevant and irrelevant modes, selecting the BLIMFs that meet the requirements for signal reconstruction. This approach improves the accuracy of relevant mode identification compared to the commonly used Bhattacharyya Distance (BD). Finally, the IWT denoising method is incorporated after signal reconstruction to further remove Gaussian white noise from the reconstructed signal, thereby enhancing the *SNR*. Simulations and experimental results verify that the proposed algorithm can effectively remove noise from echo signals and demonstrates strong robustness. It maintains good denoising performance even under low *SNR* conditions, improves the ranging accuracy of LiDAR systems, and possesses significant practical application value.

## 2. The Principle of LiDAR

**Figure 1:**
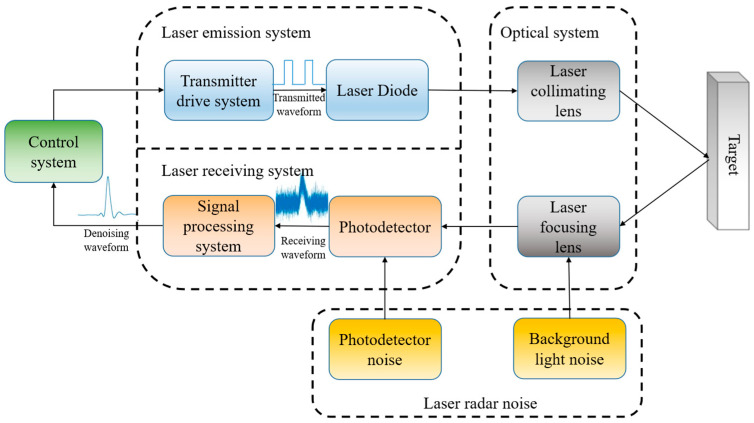
Schematic diagram of the LiDAR system.

A detailed schematic diagram of the LiDAR system utilized in this study is presented in Figure 1. The system comprises a laser emission system, a laser receiving system, an optical system, and a control system. The laser emission system emits laser pulses at a wavelength of 905 nm. The laser receiving system captures the backscattered signals from the target. Collimation and focusing of the laser beam are accomplished by the optical system. Synchronization and subsequent signal processing are executed by the control system. The pulse laser echo power equation for a planar target is [29,30]:

(1) $$P_{r}\left(t\right)=\frac{\pi D^{2}}{4R_{r}^{2}}\eta_{\mathrm{atm}}\eta_{\mathrm{sys}}\times \iint_{\sum }g\left(x,y,R\right)q\left(t\right)f_{r}\left(\varphi \right)cos\left(\varphi \right)dxdy$$

where $P_{r}\left(t\right)$ is the pulse laser echo power, D is the optical window diameter, $R_{r}$ is the distance between the lidar and the target, $\eta_{\mathrm{atm}}$ is the one-way atmospheric attenuation coefficient, $\eta_{\mathrm{atm}}=\exp\left(u\left(\lambda \right)R_{r}\right)$, $u\left(\lambda \right)$ is the atmospheric extinction coefficient, λ is the laser wavelength, $\eta_{\mathrm{sys}}$ is the system transmittance efficiency, ∑ is the cross-sectional area of the laser beam on the target surface, $f_{r}\left(\varphi \right)$ is the Lambertian target surface scattering coefficient, $f_{r}\left(\varphi \right)=\rho /\pi$, ρ is the hemispherical reflectivity of the target, φ is the beam incidence angle, $q\left(t\right)$ represents the spatial distribution of the pulsed laser, $g\left(x,y,z\right)$ represents the spatial distribution of the laser emission pulse.

In LiDAR systems, the target-backscattered echo signal is highly susceptible to interference from electronic thermal noise and background optical noise. Under real-world conditions, these noise sources are typically complex, with each exhibiting distinct probability distributions. The actual noise energy can be modeled as the sum of multiple independent random noise components. According to the Central Limit Theorem, as the number of noise sources increases, their normalized aggregate tends to approximate a Poisson distribution [31,32]. Consequently, under multi-source noise interference, the composite noise can be characterized as Poisson-distributed. This premise forms the basis for formulating the received echo signal equation in LiDAR systems:

(2) $$f\left(t\right)=x\left(t\right)+n\left(t\right)$$

In the equation, $x\left(t\right)$ represents the true echo signal, and $n\left(t\right)$ denotes the Poisson noise induced by electronic thermal noise and background optical noise. The proposed joint denoising algorithm is designed to reduce $n\left(t\right)$ and recover a high-fidelity denoised signal $x\left(t\right)$.

## 3. VMD-CPO-IWT Methodology

### 3.1. VMD Algorithm

The VMD algorithm, grounded in classical Wiener filtering, decomposes an input real-valued signal into a series of BLIMFs, each characterized by a specific center frequency [15]. To estimate the bandwidth of each BLIMF while minimizing it under the constraint that the sum of all decomposed modes equals the original signal, the following constrained variational problem is formulated:

(3) $$\underset{\left\{u_{K}\right\},\left\{\omega_{K}\right\}}{min}\left\{\sum_{k=1}^{K}{\left\|\partial_{t}\left[\left(\delta \left(t\right)+j/\pi t\right)*u_{k}\left(t\right)\right]e^{-j\omega_{k}t}\right\|}_{2}^{2}\right\}$$

(4) $$\sum_{k=1}^{K}u_{k}=f$$

where $\left\{u_{k}\right\}=\left\{u_{1},u_{2},\cdots ,u_{K}\right\}$ denotes the *k*-th mode, $\left\{\omega_{k}\right\}=\left\{\omega_{1},\omega_{2},\cdots ,\omega_{K}\right\}$ represents the center frequency of each mode, K is the number of decomposition modes, ∗ indicates the convolution operator, $\left(\delta \left(t\right)+j/\pi t\right)*u_{k}\left(t\right)$ constitutes the analytic signal via Hilbert transform, ${\left\|\partial_{t}\left[\left(\delta \left(t\right)+j/\pi t\right)*u_{k}\left(t\right)\right]e^{-j\omega_{k}^{t}}\right\|}_{2}^{2}$ quantifies the bandwidth of the *k*-th mode, and Equation (4) enforces the constraint condition for Equation (3).

To solve the constrained variational problem, a quadratic penalty term α and a Lagrangian multiplier λ are introduced. The quadratic penalty term ensures signal reconstruction fidelity, while the Lagrangian multiplier enforces strict constraint satisfaction. By integrating these components, the original constrained variational formulation is transformed into an unconstrained optimization problem, yielding the following augmented Lagrangian function:

(5) $$\begin{array}{l} L\left(\left\{u_{k}\right\},\left\{\omega_{k}\right\},\lambda \right)=\alpha \sum_{k=1}^{K}{\left\|\partial_{t}\left[\left(\delta \left(t\right)+j/\pi t\right)*u_{K}\left(t\right)\right]e^{-j\omega_{k}t}\right\|}_{2}^{2}\\ +{\left\|f\left(t\right)-\sum_{k=1}^{K}u_{K}\left(t\right)\right\|}_{2}^{2}+\langle \lambda \left(t\right),f\left(t\right)-\sum_{k=1}^{K}u_{K}\rangle \end{array}$$

Herein, the weighting coefficient of the quadratic penalty term α is typically derived from the Bayesian prior distribution of additive independent and identically distributed (i.i.d.) Gaussian noise that exhibits an inverse proportionality to the noise level in the signal. The quadratic penalty term α ensures reconstruction fidelity under Gaussian noise contamination, whereas the Lagrangian multiplier λ guarantees strict enforcement of the constraint condition.

Consequently, the minimization problem in Equation (3) transforms into a saddle point search for the augmented Lagrangian function in Equation (5). VMD employs the Alternating Direction Method of Multipliers (ADMM) to solve this problem through iterative local optimizations. The preliminary update forms for $\left\{u_{k}\right\}$, $\left\{\omega_{k}\right\}$, and λ are respectively given by [15]:

(6) $${\hat{u}}_{k}^{n+1}\left(\omega \right)=\frac{\hat{f}\left(\omega \right)-\sum_{i\neq k}{\hat{u}}_{i}\left(\omega \right)+\frac{\hat{\lambda }\left(\omega \right)}{2}}{1+2\alpha {\left(\omega -\omega_{k}\right)}^{2}}$$

(7) $$\omega_{k}^{n+1}=\frac{\int_{0}^{+\infty }\omega {\left|{\hat{u}}_{k}\left(\omega \right)\right|}^{2}d\omega }{\int_{0}^{+\infty }{\left|{\hat{u}}_{k}\left(\omega \right)\right|}^{2}d\omega }$$

(8) $${\hat{\lambda }}^{n+1}\left(\omega \right)={\hat{\lambda }}^{n}\left(\omega \right)+\tau \left(\hat{f}\left(\omega \right)-\sum_{k=1}^{K}{\hat{u}}_{k}^{n+1}\left(\omega \right)\right)$$

The algorithm proceeds iteratively via Equations (4)–(6) until either the convergence criterion is satisfied or the maximum iteration count is reached, at which point the iteration terminates. The convergence condition requires satisfaction of the following inequality constraint:

(9) $$\sum_{k=1}^{K}{\left\|{\hat{u}}_{k}^{n+1}-{\hat{u}}_{k}^{n}\right\|}_{2}^{2}/{\left\|{\hat{u}}_{k}^{n}\right\|}_{2}^{2}<\varepsilon$$

where ${\hat{u}}_{k}$ enotes the Fourier spectrum of the *k*-th mode, $\omega_{k}$ represents the center frequency of each mode, $\hat{f}\left(\omega \right)$ signifies the Fourier spectrum of the original signal, τ is the noise tolerance parameter, ε denotes the convergence criterion tolerance (generally set to $10^{-6}$), and n indicates the iteration count.

### 3.2. CPO Algorithm

The CPO is a novel metaheuristic algorithm inspired by the distinctive defense mechanisms of crested porcupines. It dynamically adjusts search strategies through dual-mode operations—”Threat Perception” and “Defensive Tactics”—enabling rapid convergence toward potential optima during early stages while facilitating refined local exploitation in later phases [33]. CPO addresses multi-dimensional optimization problems where each dimension corresponds to a minimization or maximization objective. The algorithm initiates by randomly initializing dimensions within the problem-specific search space, subsequently optimizing solutions through Equation (10):

(10) $$\vec{X_{i}}=\vec{L}+\vec{r}\times \left(\vec{U}-\vec{L}\right),i=1,2,\cdots ,N$$

where N denotes the population size (i.e., total number of individuals), $\vec{X_{i}}$ represents the *i*-th candidate solution in the search space, $\vec{L}$ and $\vec{U}$ are the lower and upper boundaries of the search range, respectively, and $\vec{r}$ is a uniformly distributed random vector initialized between 0 and 1.

When employing the CPO to determine optimal VMD parameters, the operational procedure comprises the following steps:

(i) Global Exploration Phase

During this phase, the crested porcupine detects predators at considerable distance, deploying dual visual and auditory deterrent mechanisms.

Primary Defense Strategy: The porcupine erects and flutters its quills as a visual warning to predators. This behavior is mathematically modeled by the position update equation:

(11) $$\vec{x_{i}^{t+1}}=\vec{x_{i}^{t}}+\tau_{1}\times \left|2\times \tau_{2}\times \vec{x_{CP}^{t}}-\vec{y_{i}^{t}}\right|$$

where $\vec{x_{CP}^{t}}$ denotes the optimal solution of the function that determines the value of *t*, $\vec{y_{i}^{t}}$ is a vector generated between the current crested porcupine (CP) and the population CP, and denotes the position of the predator at the first iteration. $\tau_{1}$ is a random number drawn from a normal distribution, and $\tau_{2}$ is a random value in the interval [0, 1].

Secondary Defense Strategy: The crested porcupine generates acoustic threats to further deter predators, mathematically modeled by the position update:

(12) $$\vec{x_{i}^{t+1}}=\left(1-\vec{U_{1}}\right)\times \vec{x_{i}^{t}}+\vec{U_{1}}\times \left(\vec{y}+\tau_{3}\times \left(\vec{x_{r1}^{t}}-\vec{x_{r2}^{t}}\right)\right)$$

where r1 and r2 are two random integers drawn from the range [1, *N*], and $\tau_{3}$ are random values generated between 0 and 1.

(ii) Local Exploitation Phase

At this stage, predators are close to the crested porcupine, which will deploy olfactory and physical attacks against them.

Third Defense Strategy: The crested porcupine secretes a foul-smelling substance that diffuses through the surrounding area to prevent predators from advancing closer. Its mathematical model is:

(13) $$\vec{x_{i}^{t+1}}=\left(1-\vec{U_{1}}\right)\times \vec{x_{i}^{t}}+\vec{U_{1}}\times \left(\vec{x_{r1}^{t}}+S_{i}^{t}\times \left(\vec{x_{r2}^{t}}-\vec{x_{r3}^{t}}\right)-\tau_{3}\times \vec{\delta }\times \gamma_{t}\times S_{i}^{t}\right)$$

where r3 is a random integer within [1, *N*], $\vec{\delta }$ denotes the parameter controlling search direction, $\vec{x_{i}^{t+1}}$ represents the position of the *i*-th individual at iteration *t*, $\gamma_{t}$ is the defense factor, $\tau_{3}$ is a random value in [0, 1], $S_{i}^{t}$ signifies the odor diffusion factor.

Fourth Defense Strategy: When all preceding strategies fail, the crested porcupine launches physical attacks against predators. Its mathematical model is:

(14) $$\vec{x_{i}^{t+1}}=\vec{x_{CP}^{t}}+\left(\alpha \left(1-\tau_{4}\right)+\tau_{4}\right)\times \left(\delta \times \vec{x_{CP}^{t}}-\vec{x_{i}^{t}}\right)-\tau_{5}\times \delta \times \gamma_{t}\times \vec{F_{i}^{t}}$$

where $\vec{x_{CP}^{t}}$ denotes the global optimum solution, $\vec{x_{i}^{t}}$ represents the position of the *i*-th individual at iteration *t*, signifies the predator position at the corresponding location, $\tau_{4}$ and $\tau_{5}$ are random variables uniformly distributed in [0, 1], $\vec{F_{i}^{t}}$ quantifies the inelastic collision force generated during physical assault.

### 3.3. Minimum Average Envelope Entropy (MAEE)

LiDAR echo signals demonstrate distinctive non-stationary and time-varying properties, challenging the efficacy of conventional fitness functions under complex operational conditions. Information entropy serves as a robust metric for evaluating signal sparsity, where higher entropy values directly indicate greater signal uncertainty. To enhance sparsity characterization, envelope entropy is introduced as an advanced measure that more accurately quantifies the sparse nature of raw signals [34].

The average envelope entropy (AEE) is defined as the mean value of envelope entropy across all variational mode components obtained through VMD under specific parameters K and α. The AEE metric is employed as the fitness function during the CPO optimization process, with the MAEE serving as the ultimate optimization objective. This approach thereby optimizes the VMD parameters K and α, as formalized in Equation (15).

(15) $$\langle \hat{K},\hat{\alpha }\rangle =\underset{\langle K,\alpha \rangle }{argmin}\left\{\frac{1}{\hat{K}}\sum_{i=1}^{\hat{K}}H_{en}\left(i\right)\right\}$$

where $\hat{K}$ and $\hat{\alpha }$ denote the optimal parameters, $H_{en}\left(i\right)$ represents the envelope entropy of each variational mode component, which can be computed via Equations (16) and (17).

(16) $$H_{en}\left(j\right)=-\sum_{j=1}^{N}p_{j}log_{2}\left(p_{j}\right)$$

(17) $$p_{j}=a\left(j\right)/\sum_{j=1}^{N}a\left(j\right)$$

where N is the sampling point, and $p_{j}$ is the normalized value of the envelope $a_{j}$.

### 3.4. VMD-CPO Algorithm

To optimize the key parameters of VMD, we integrate the Crested Porcupine Optimizer, forming the VMD-CPO algorithm. The modal component number K and the quadratic penalty factor α have a significant impact on the decomposition results of VMD. When K is too small, the signal decomposition may be incomplete, and some components may overlap with other modes. When K is too large, over-decomposition and mode repetition may occur. When α is too small, the bandwidth of the decomposed modes may be too large, and some modes may overlap with others. When α is too large, the bandwidth of the decomposed modes may be too small, and some modes of the original signal may be lost [35]. Therefore, the adaptability of the variational modal decomposition algorithm lies in how to obtain the optimal values of parameters K and α.

**Figure 2:**
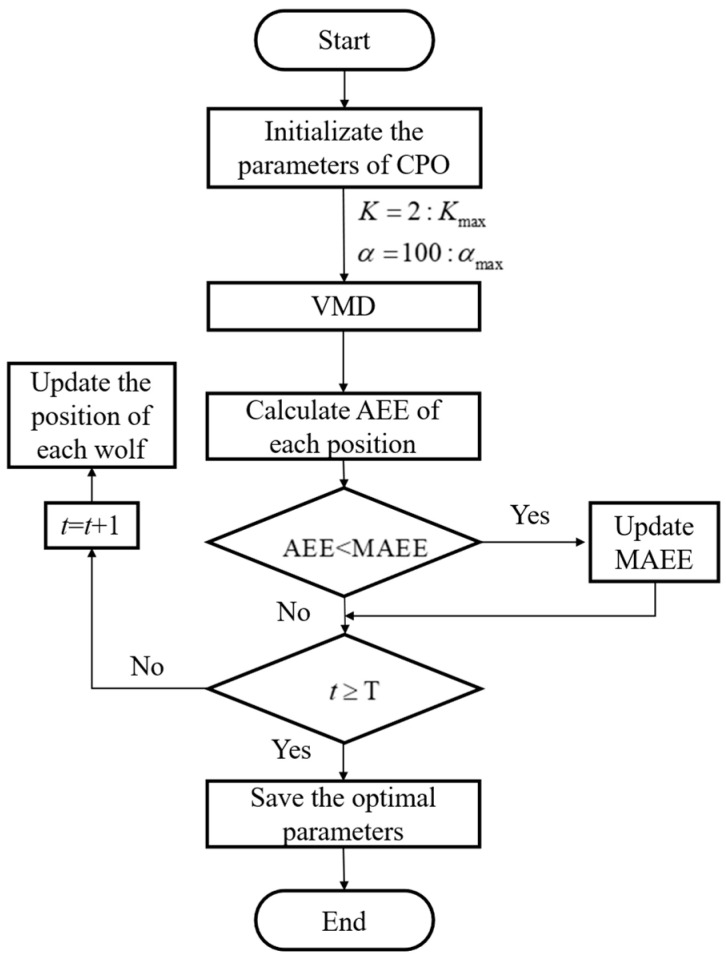
Flowchart of the proposed VMD-CPO.

Based on CPO and MAEE, the details of the VMD-CPO algorithm are summarized as follows, with the process shown in Figure 2.

Step 1: Initialize the CPO parameters $K_{max}$ and $\alpha_{max}$. Here, $K_{max}$ is set to 13 and $\alpha_{max}$ is set to 8000 to avoid incomplete signal decomposition.

Step 2: Perform VMD calculations on LiDAR echo signals.

Step 3: Calculate the AEE for each position. When the AEE is lower than the current optimal fitness value, update the MAEE.

Step 4: Determine whether to terminate the iteration. If t is less than T (the maximum number of iterations), then let t=t+1, and update the position of the crested porcupine population. Otherwise, terminate the iteration and save the optimal parameters.

### 3.5. WD

According to Bayesian theory, probability density functions (PDFs) can reflect differences between signal distributions [25]. Therefore, we utilize kernel smoothing density functions to estimate the PDFs of each mode and the input signal. Relevant modes and irrelevant modes are distinguished by computing their similarity.

Commonly used similarity measurement methods can be divided into two categories: (1) the information-theoretic measures, including the Kullback–Leibler divergence (KLD), and (2) the geometric distance measures such as the Hausdorff distance, BD, etc. [36]. Reference [25] compares information-theoretic measures with geometric distance measures and demonstrates that the latter are more effective than the former. Comprehensively considering these factors, this paper adopts the WD as the similarity measurement method to distinguish relevant and irrelevant modes. The WD is an indicator measuring the similarity between two distributions, whose advantage lies in its ability to reflect the distance between distributions even when their support sets have no overlap or minimal overlap, while also being sensitive to outliers [37]. The calculation formula for WD is:

(18) $$W\left(p,q\right)=\underset{\gamma \sim \prod \left(p,q\right)}{inf}E_{x,y\sim \gamma }\left[\left\|x-y\right\|\right]$$

where $\prod \left(p,q\right)$ is the set of all possible joint probability distributions of the marginal distributions of different data p and q. Sampling $\left(x,y\right)\sim \gamma$ from each possible joint probability distribution γ yields a pair of samples x and y. Calculating the distance $\left\|x-y\right\|$ between this pair of samples yields the corresponding expected value $E_{x,y\sim \gamma }\left[\left\|x-y\right\|\right]$. The lower bounds obtained for the expected value in the joint probability distribution γ are WD.

The similarity measurement $L\left(i\right)$ between the input signal f and each mode $\mathrm{BLIM}F_{i}$ is defined as follows:

(19) $$L\left(i\right)=WD\left[pdf\left(f\right),pdf\left(BLIMF_{i}\right)\right]$$

Relevant modes can be determined by evaluating the slope of distances between two adjacent PDFs and the input signal. The maximum slope θ can be defined as:

(20) $$\theta =max\left|L\left(i+1\right)-L\left(i\right)\right|,i=1,2,\cdots ,\left(N-1\right)$$

The boundary index between relevant modes and irrelevant modes is defined as follows:

(21) $$K_{r}=i$$

### 3.6. IWT

Compared with the hard thresholding method, the denoised signal processed by the soft thresholding method exhibits better smoothness [38]. Therefore, this paper adopts the soft thresholding technique to recover useful signals concealed by noise:

(22) $${\tilde{d}}^{i}\left(t\right)=\left\{\begin{array}{ll} sgn\left(d^{i}\left(t\right)\right)\left(d^{i}\left(t\right)-T_{i}\right), & \left|d^{i}\left(t\right)\right|>T_{i}\\ 0, & \left|d^{i}\left(t\right)\right|\leq T_{i} \end{array}\right.$$

where $d^{i}\left(t\right)$ represents the wavelet coefficients after decomposition, and $T_{i}$ denotes the threshold.

Threshold selection is critical for wavelet denoising. Compared with the VisuShrink thresholding method [39] and the Rigsure thresholding method [40], the improved Stein’s unbiased risk estimate (ISURE) proposed in this paper offers a wider optimization range for optimal thresholds and exhibits reduced dependence on data volume.

Let $Y={\left[y_{0},y_{1},\cdots ,y_{N}\right]}^{T}$ represent the noisy data to be denoised, $s_{i}$ denote the pure signal data, $\hat{s}$ is the threshold-processed data, with signal length N. The mapping function $g\left(Y\right)$ from the noisy data Y to $R^{N}$ is defined as follows:

(23) $$g\left(Y\right)=\hat{s}-Y$$

According to the principle of Stein’s unbiased risk estimate (SURE) [41], the estimated mean squared error (MSE) is derived as follows:

(24) $$E\left(MSE\right)=E\left[\sum_{i=1}^{N}{\left(\hat{s}-s_{i}\right)}^{2}/N\right]=N+{\left\|g\left(Y\right)\right\|}^{2}+2\sum_{i=1}^{N}\left(\frac{\partial g_{i}}{\partial y_{i}}\right)$$

Here, Equation (25) is derived from Equation (23).

(25) $$g_{i}=\eta \left(y_{i},T_{i},m\right)-y_{i}$$

Substituting into Equation (24), the partial derivative of $E\left(Y_{MSE}\right)$ with respect to $T_{i}$ is computed to obtain the gradient value. The optimal threshold is then derived by optimizing the mean squared error estimate using gradient descent. However, Equation (24) exhibits significant limitations: it applies identical threshold values across all levels during multilevel wavelet decomposition, failing to adapt to multi-scale characteristics [42]. Therefore, an improved method is proposed by generalizing the approach, substituting the data values Y with wavelet coefficients as variables.

Let the high-frequency wavelet coefficients at the j-th level of the signal to be denoised be denoted as $u={\left[u_{1},u_{2},\cdots ,u_{M_{j}}\right]}^{T}$. Among these, the high-frequency wavelet coefficients of the pure signal are $v_{j}$, and the high-frequency wavelet coefficients of the denoised signal are ${\hat{v}}_{j}$. $g\left(u\right)$ The mapping function from the noisy wavelet coefficients u to the denoised coefficients $R^{M_{j}}$, and its formula is expressed as:

(26) $$g\left(u\right)=\hat{v}-u=\eta \left(u,\lambda ,m\right)-u$$

**Lemma** **1**. *Let* $X\sim N\left(0,1\right)$ *be a real-valued random variable, and consider an estimator* $X+h\left(X\right)$ *of* ξ. *Suppose the mapping h:* R→R *is almost everywhere differentiable and satisfies, where* $\nabla_{i}=\frac{\partial }{\partial X_{i}}$*. Then for every*$i\in \left\{1,2,\cdots ,p\right\}$*, the following equality holds:*

(27) $$E_{\xi }{\left\|X+h\left(X\right)-\xi \right\|}^{2}=p+E_{\xi }\left\{{\left\|h\left(X\right)\right\|}^{2}+2\nabla h\left(X\right)\right\}$$

Substituting u and v from Equation (26) into X, ξ and $h\left(X\right)$ in Equation (27), respectively, we finally obtain an estimate of $M_{j}$ times the MSE deviation of the denoised coefficients and the pure signal coefficients:

(28) $$E\left[\sum {\left(\hat{v}-v\right)}^{2}\right]=E\left[\sum {\left(u+g\left(u\right)-v\right)}^{2}\right]=M_{j}\sigma^{2}+\sum \left[g{\left(u\right)}^{2}+2\sigma^{2}\frac{\partial g\left(u\right)}{\partial \left(u\right)}\right]$$

The value of $T_{i}$ that minimizes Equation (27) for each layer is the threshold. This improvement provides a specific, accurate, and simple function expression for the last partial derivative in the estimation Equation (28), which facilitates the selection of optimization algorithms.

### 3.7. VMD-CPO-IWT Algorithm

**Figure 3:**
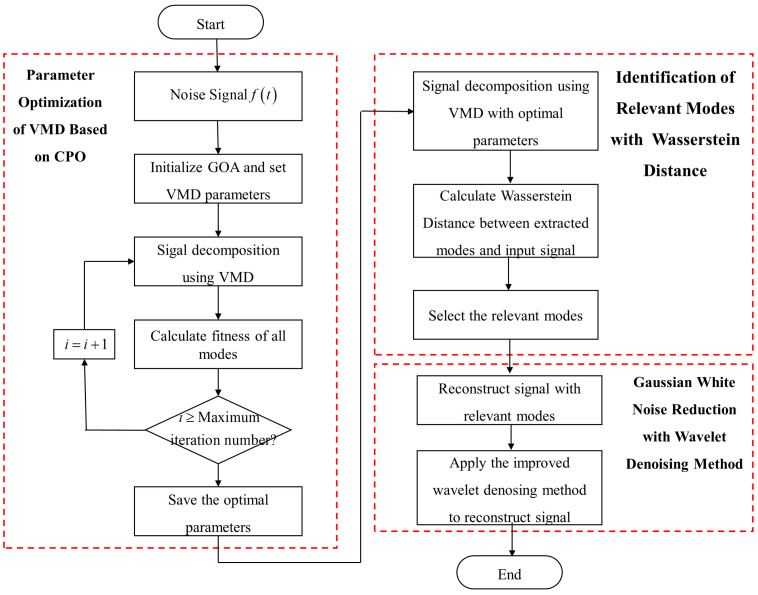
Flowchart of the VMD-CPO-IWT denoising algorithm.

Figure 3 illustrates the flowchart of the VMD-CPO-IWT algorithm. The specific procedural steps are as follows:

Step 1: Input the noisy original LiDAR echo signal $f\left(t\right)$.

Step 2: Set the optimization ranges for VMD parameters. Here, the range for the number of modes K is [2, 15], and the range for the quadratic penalty factor α is [200, 10,000]. Additionally, initialize the CPO model parameters: maximum iterations $t_{max}=30$, initial population size $N_{p}=50$.

Step 3: Decompose the signal using VMD. Calculate the fitness value for each search position. Save the minimum fitness value from each iteration.

Step 4: Iterate cyclically until reaching the maximum iterations, i.e., check if $i\geq t_{max}$. If yes, terminate the iteration. Otherwise, set i=i+1 and continue iterating. Save the optimal parameters and best fitness value.

Step 5: Decompose the signal with VMD using the optimal parameters.

Step 6: Calculate the PDF of each mode and the input signal to obtain their WD. Screen relevant modes and reconstruct the signal.

Step 7: Perform IWT denoising on the reconstructed signal to further filter out Gaussian white noise.

## 4. Results

### 4.1. Evaluation Indicators

This paper employs the *SNR* and *RMSE* as metrics to evaluate the effectiveness of the proposed denoising algorithm. A higher *SNR* and a smaller *RMSE* indicate superior denoising performance. *SNR* measures the level of the desired signal relative to the background noise, while *RMSE* quantifies the discrepancy between model-predicted values and actual observed values. These metrics are essential for assessing the accuracy and efficiency of denoising algorithms in signal processing. The definitions of *SNR* and *RMSE* are as follows:

(29) $$SNR=10\log\left[\frac{\sum_{i=1}^{N}S_{\mathrm{original}}^{2}}{\sum_{i=1}^{N}{\left(S_{\mathrm{original}}-S_{\mathrm{re}}\right)}^{2}}\right]$$

(30) $$RMSE=\sqrt{\frac{1}{N}\sum_{i=1}^{N}{\left[S_{\mathrm{original}}-S_{\mathrm{re}}\right]}^{2}}$$

where $S_{\mathrm{original}}$ is the original signal, $S_{\mathrm{re}}$ is the reconstructed signal, and N is the signal length.

### 4.2. Experiments with Simulated Signals

**Figure 4:**
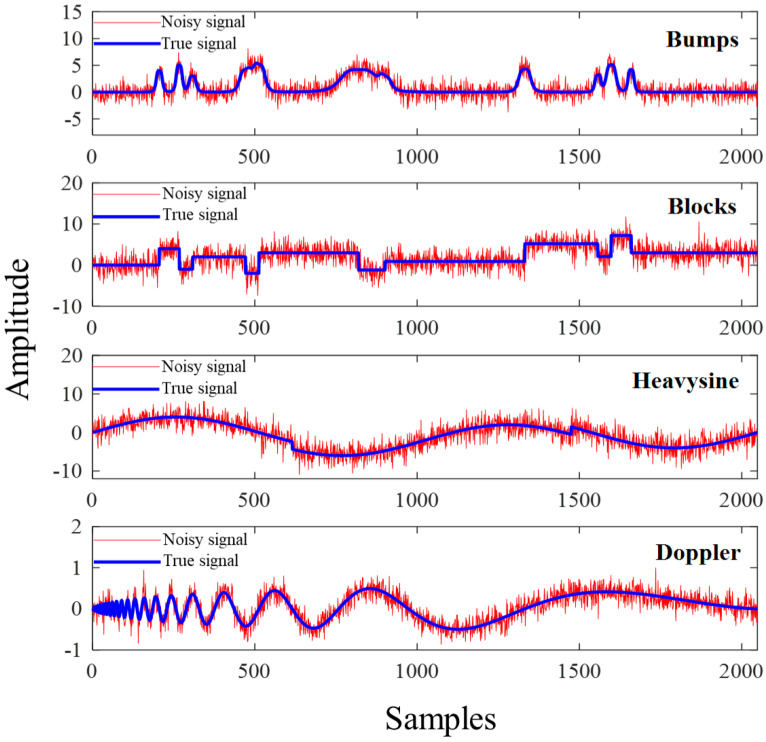
Four typical signals (*SNR_in_* = 4 dB).

To visually compare the performance of different algorithms under challenging conditions, we selected four typical nonlinear non-stationary signals: Bumps, Blocks, Heavysine, and Doppler signals. Gaussian white noise was added to each signal with an input *SNR* (*SNR_in_*) of 4 dB and signal length of *N* = 2048. Furthermore, to comprehensively evaluate the algorithms’ robustness, we will explore their performance across a broader *SNR_in_* range (−5 dB to 10 dB) in subsequent sections. as illustrated in Figure 4. The blue and red lines represent the true signal and noisy signal, respectively. Through simulation experiments, the proposed VMD-CPO-IWT method was compared with the NeighCoeff-db4 wavelet method (WT-db4) [42], the detrended fluctuation analysis thresholded EMD-based denoising method (EMD-DFA) [43], and VMD combined with the whale optimization algorithm (VMD-WOA) [27]. Using *SNR* and *RMSE* metrics, the efficiency and reliability of the improved algorithm were evaluated, and its denoising capability was comprehensively analyzed.

**Figure 5:**
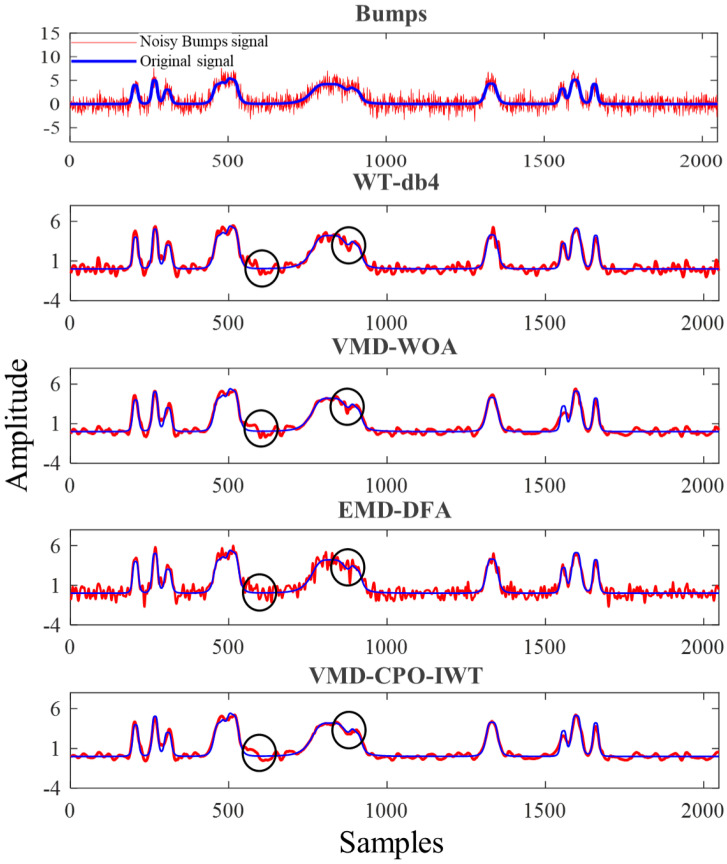
Denoising results of noisy Bump signals using different methods (*SNR_in_* = 4 dB).

For the Bumps signal with *SNR_in_* = 4 dB, the comparative results of different denoising methods are shown in Figure 5. The blue line and red line in the figure represent the original and noisy Bumps signal, respectively. It can be observed that the proposed VMD-CPO-IWT algorithm achieves the best denoising performance. The processed signal is smoother and exhibits the highest similarity to the original signal, particularly in the regions marked by circles. The EMD-DFA algorithm demonstrates the poorest performance, with the denoised signal showing numerous “burrs”. Although WT-db4 and VMD-WOA methods yield better denoising results compared to EMD-DFA, the denoised signals still lose significant useful components and exhibit substantial distortion at the beginning, end, and wave peak regions of the denoised signal.

Table 1 lists the output *SNR* (*SNR_out_*) and *RMSE* values for the Bumps signal with *SNR_in_* = 4 dB. The results show that the VMD-CPO-IWT method achieves the highest *SNR_out_* (17.25 dB) and the lowest *RMSE* (0.1736), indicating that compared to the other three methods, the VMD-CPO-IWT method has stronger noise reduction capability for the input signal. Both Figure 5 and Table 1 confirm that the proposed method outperforms other methods in denoising and preserving signal integrity.

**Figure 6:**
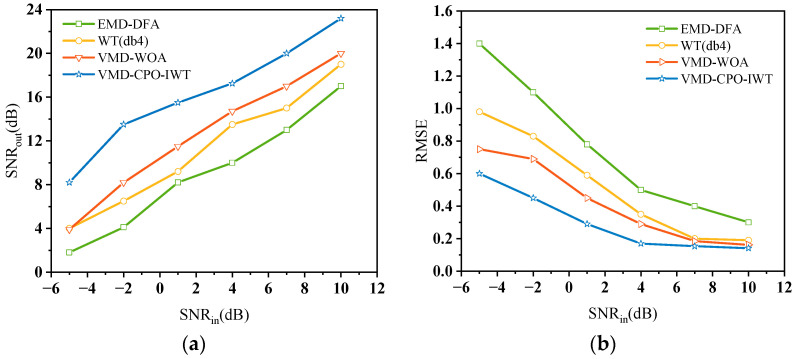
(**a**) *SNR_out_* of Bumps signal after denoising at different *SNR_in_*; (**b**) *RMSE* of Bumps signal after denoising for different *SNR_in_*.

Figure 6 shows the denoising performance of different algorithms for Bumps signals with *SNR_in_* ranging from −5 dB to 10 dB. It can be seen that compared to the other four algorithms, the proposed algorithm achieves the best denoising performance under different *SNR_in_*. When *SNR_in_* = 10 dB, the *SNR_out_* can reach up to 23.22 dB, with a corresponding *RMSE* of only 0.1412. Even in the low *SNR* case of *SNR_in_* = −5 dB, the *SNR_out_* of signals processed by VMD-CPO-IWT can still reach 8.23 dB, while the *SNR_out_* of the other four algorithms are all less than 4.5 dB. Furthermore, comparative results of *RMSE* under different *SNR_in_* demonstrate that the VMD-CPO-IWT algorithm can effectively retain useful information in the original signal.

**Figure 7:**
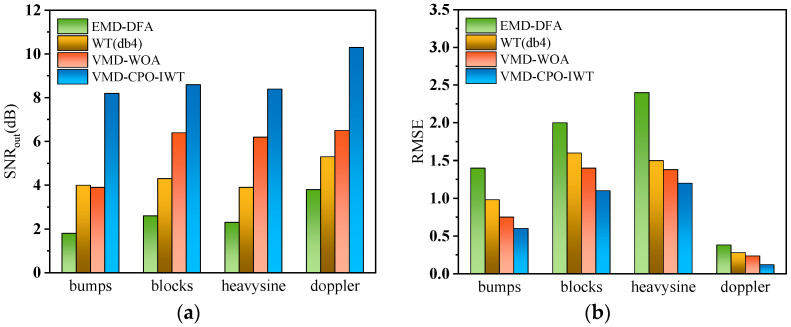
(**a**) *SNR_out_* of four synthetic noisy signals after denoising at *SNR_in_* = −5 dB; (**b**) *RMSE* of four synthetic noisy signals after denoising at *SNR_in_* = −5 dB.

To test the denoising performance of different algorithms on various noisy signals, we additionally added three synthetic signals: Blocks, Heavysine, and Doppler signals, with *SNR_in_* set to −5 dB. The results are shown in Figure 7. It can be seen that the VMD-CPO-IWT algorithm achieves optimal denoising performance across all four test signals, obtaining an average *SNR* of 8.9 dB. Particularly for the denoised Doppler signal, it attains the maximum *SNR_out_* value of 10.3 dB while having the smallest *RMSE* of only 0.118. Conversely, the EMD-DFA algorithm demonstrates the worst denoising performance, with its obtained *SNR* being lower than the other four algorithms across all test signals. Although VMD-WOA outperforms WT (db4) and EMD-DFA, it still cannot match the VMD-CPO-IWT algorithm. Based on the above analysis, the proposed VMD-CPO-IWT algorithm can achieve relatively good denoising and detail preservation performance in low *SNR* scenarios, and possesses excellent robustness.

Computational complexity serves as a critical metric for evaluating the practical utility of algorithms. For this purpose, we statistically analyzed the runtime for processing the same Bumps signal (*N* = 2048, *SNR*_in_ = 4 dB) using MATLAB R2023a on a computer configured with an Intel Core i7-12700H CPU and 16 GB RAM. The results are presented in Table 2, which shows that the proposed CPO-VMD-IWT algorithm requires a longer execution time than the other compared methods. This is primarily attributed to its core optimization process: the CPO algorithm needs to iteratively evaluate candidate parameter combinations represented by multiple individuals, with each evaluation requiring a complete VMD to compute the MAEE. This structure of a “decomposition loop nested within an optimization loop” inevitably introduces a high computational load. In comparison, the WT-db4 and EMD-DFA methods do not involve parameter optimization steps, thus achieving faster computational speeds. The VMD-WOA method also incurs relatively high computational costs due to its multiple VMDs during the optimization process. However, it is important to emphasize that the increased computational cost of the proposed algorithm is traded for a significant improvement in denoising performance, particularly in scenarios with extremely low *SNR*_in_.

### 4.3. Performance Improvement Based on the Modified Algorithm

The proposed VMD-CPO-IWT algorithm is a joint algorithm composed of three parts. We conducted the following simulations to confirm the performance contributions of each part compared with other methods.

#### 4.3.1. Better Denoising Performance Based on CPO Optimization

The decomposition performance of VMD is significantly influenced by the configuration of its key parameters, thus requiring the assistance of optimization algorithms for parameter tuning. Among numerous optimizers, CPO, PSO, and WOA are all commonly used options. However, CPO demonstrates notable advantages in terms of stability, global optimization capability, and sensitivity to local optima. Specifically, PSO primarily relies on individual and global historical best positions to guide particle movement. Its search mechanism is relatively singular, which can easily lead to decreased population diversity and entrapment in local optima [22]. WOA conducts searches by simulating limited behaviors of whales, such as encircling prey and spiral updating. Its search patterns are relatively fixed and lack flexibility, making it difficult to adapt to complex and variable parameter spaces [23]. In contrast, CPO can comprehensively explore the parameter space from multiple perspectives using different strategies, not only increasing the probability of discovering the global optimum but also exhibiting stronger robustness. Furthermore, the defensive search mechanism introduced by CPO enables it to quickly adjust the search direction and effectively escape local optimum regions even when trapped, thereby ensuring stability and convergence efficiency during extended optimization processes [44].

**Figure 8:**
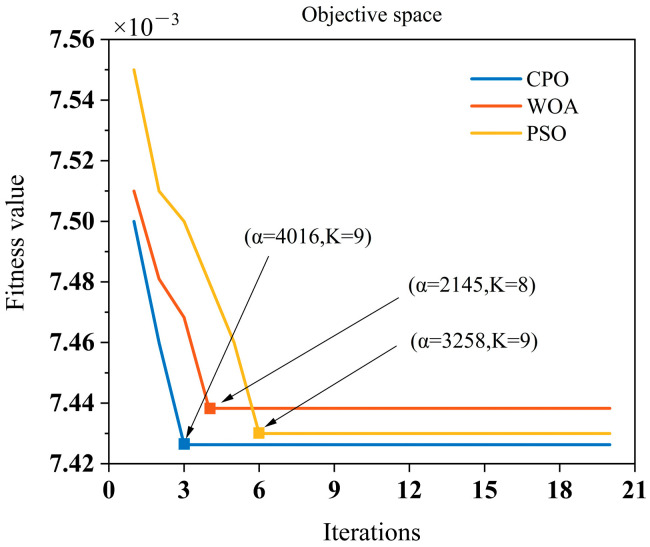
Convergence curves of CPO, WOA, and PSO when optimizing VMD parameters.

To intuitively illustrate the issue, we conducted a simulated comparison among CPO, PSO, and WOA. The objective was to compare their performance in optimizing VMD parameters. The AEE was used as the objective function, with a Bumps signal contaminated by 4 dB Gaussian white noise serving as the test signal. Comparative results are shown in Figure 8. It can be observed that CPO exhibits faster convergence speed compared to the other two algorithms. CPO converges at the third iteration, while PSO and WOA converge at the fourth and sixth iterations, respectively. Furthermore, CPO achieves a lower AEE value. The optimal VMD parameters obtained through CPO, WOA, and PSO iterations are $\left(\alpha =4016,K=9\right)$, $\left(\alpha =2145,K=8\right)$, and $\left(\alpha =3258,K=9\right)$, respectively.

Based on the optimal parameters obtained above, simulations were conducted for the CPO-based, PSO-based, and WOA-based methods to test their performance. The main steps of these methods are as follows. First, decompose the test signal using the optimal parameters obtained by CPO, PSO, and WOA. Then, utilize WD to select relevant modes and further denoise the signal with IWT after reconstruction. Simulation results are listed in Table 3. Compared with PSO and WOA, the CPO-based method achieves a higher *SNR_out_* of 15.792 dB for the processed signal. Additionally, it obtains a lower *RMSE* of 0.2987. Therefore, the above results prove that CPO is superior to PSO and WOA in optimizing VMD parameters.

#### 4.3.2. Mode Identification Based on WD

To validate the effectiveness of the WD, we compare it with the BD, which measures the distance between two discrete or continuous probability distributions and serves as an effective approach to demonstrate similarity [23].

For discrete probability distributions p and q defined on the same domain, BD is defined as:

(31) $$BD\left(p,q\right)=-ln\left(BC\left(p,q\right)\right)$$

where $BC\left(p,q\right)=\sum_{x}\sqrt{p\left(x\right)q\left(x\right)}$ denotes the Bhattacharyya coefficient.

**Figure 9:**
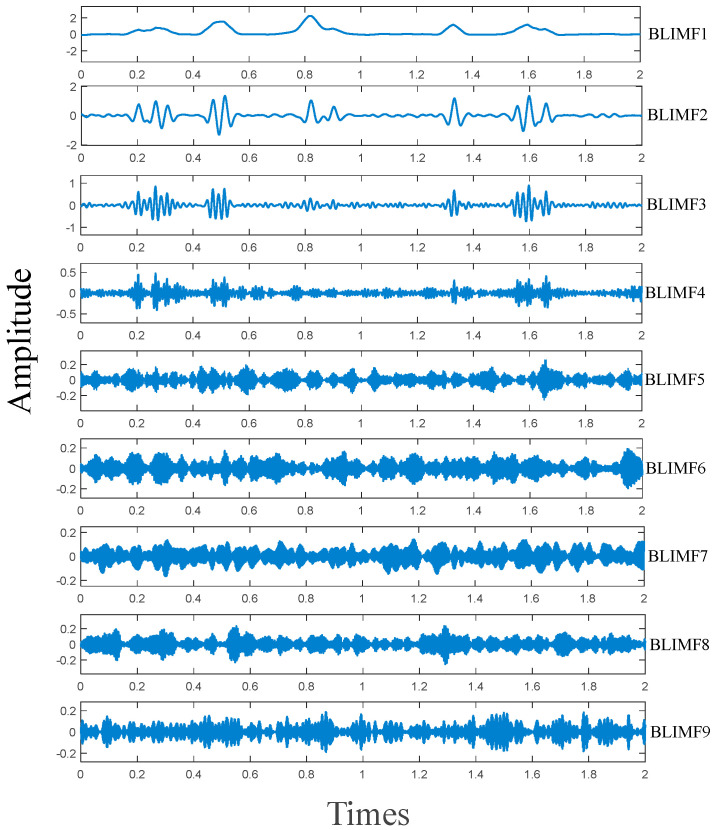
VMD results of noisy Bumps signal (*SNR_in_* = 7 dB).

**Figure 10:**
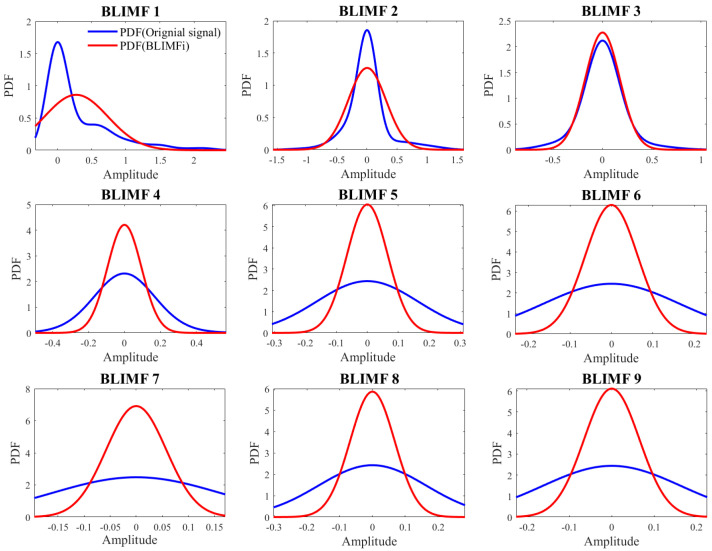
Superposition of the PDF of the Bumps signal and those of its modes (*SNR_in_* = 7 dB).

We use the Bumps signal contaminated by Gaussian white noise as the test signal, with *SNR_in_* = 7 dB. Before simulation, VMD decomposes the test signal into multiple modes and obtains the PDF of each mode and the test signal via the kernel density estimation method. Subsequently, BD and WD are applied to calculate the distances between the test signal and each mode. The decomposition modes of the test signal are shown in Figure 9. Which displays nine modes from low to high frequency. BLIMF1 contains most of the original signal, though some signal details are missing. However, BLIMF2 and BLIMF3 compensate for this signal loss. Other BLIMFs exhibit noise waveforms with no discernible useful signal. Figure 10 shows the PDFs of the noisy Bumps signal and its BLIMFs. As illustrated, BLIMF1~BLIMF3 show the highest similarity to the original signal, with the most analogous curve shapes. Additionally, BLIMF4~BLIMF9 exhibit significant differences from the original signal, which corresponds to the simulation results in Figure 9.

**Figure 11:**
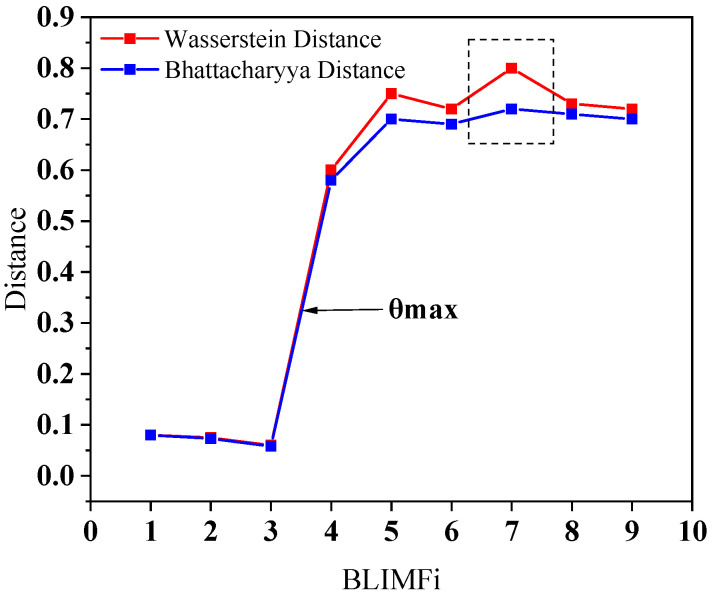
WD and BD versus the BLIMF index of the test signal.

Figure 11 shows the distance distributions between each BLIMF and the original signal. It can be observed that the largest distance difference occurs between BLIMF3 and BLIMF4, indicating that both WD and BD correctly identify the relevant modes (BLIMF1, BLIMF2, BLIMF3). According to Figure 10, the similarity between BLIMF7 and the test signal is greater than that between BLIMF6 and the test signal. However, the BD curve in Figure 11 is misleading. The above results demonstrate that WD outperforms BD in measuring similarity between signals, as WD consistently obtains the correct correlations among these modes.

#### 4.3.3. Performance Enhancement Achieved by the Improved Wavelet Threshold Method

After reconstructing the signal by selecting relevant modes, further denoising is required via IWT. This subsection validates whether IWT can reduce Gaussian white noise and improve *SNR* when processing short signals, which is crucial. As analyzed earlier, the key to wavelet denoising performance lies in threshold selection, whose core is balancing thorough noise removal and data integrity. Therefore, this section conducts comparative simulations on denoising effects for signals of varying lengths using wavelet filtering algorithms: VisuShrink, Rigsure, and ISURE. The test signal is a Heavysine signal contaminated by Gaussian white noise. First, decompose the original signal via VMD. Then apply WD to BLIMFs to select relevant modes. Subsequently, process the reconstructed signal with three different threshold-based wavelet denoising methods and perform denoising performance analysis.

**Figure 12:**
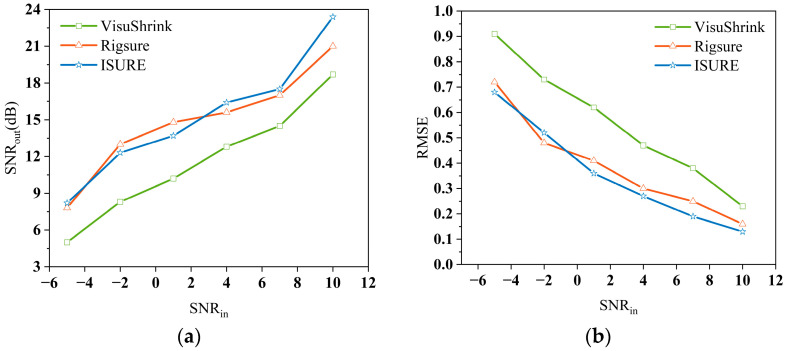
(**a**) *SNR_out_* of using three threshold methods at *N* = 150 ns; (**b**) *RMSE* of using three threshold methods at *N* = 150 ns.

Simulation results are shown in Figure 12. For long signals (*N* = 150 ns), the denoising effects of Rigsure and ISURE show little difference, with the improved algorithm offering only slight enhancement, while VisuShrink is significantly inferior to the other two methods. During close-range detection, effective LiDAR echo data constitutes only a small portion of the full echo dataset. Moreover, excessively long invalid data affects processing efficiency without improving denoising accuracy. Therefore, subsequent simulations adopt effective waveform segments with data length less than 100 ns, applying three threshold methods to test signals of varying lengths using multi-level wavelet decomposition.

Liu et al. discovered that when the decomposition level is too high, the power-law decrease in wavelet coefficient length causes significant data distortion in VisuShrink and Rigsure at high decomposition levels [45]. Therefore, to minimize data distortion while maximizing denoising extent, a 3-level wavelet decomposition with “sym4” was selected for simulation experiments. The *RMSE* values of the three threshold methods under various simulation data are listed in Table 4. Simulations reveal that all methods depend on sample size, meaning longer data yields higher average denoising accuracy. Among them, ISURE achieves two orders of magnitude higher average denoising accuracy than VisuShrink at shorter lengths, and slightly outperforms Rigsure, indicating its average denoising accuracy is less affected by signal length. VisuShrink delivers the poorest denoising performance, followed by Rigsure. However, all three exhibit comparable average denoising accuracy for long signals.

### 4.4. Experiments on a LiDAR Echo Signal

**Figure 13:**
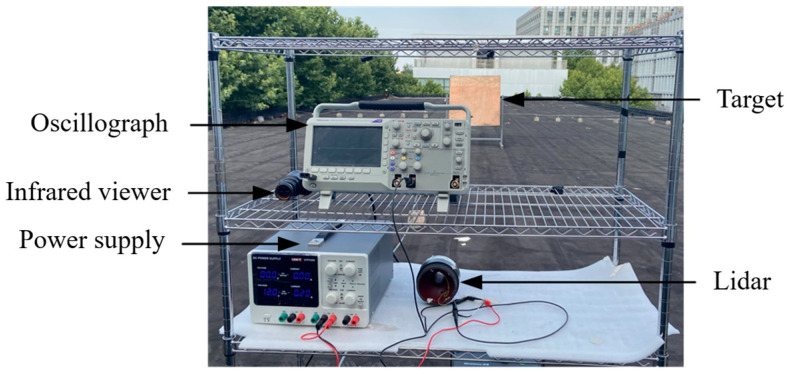
Experimental setup.

Actual LiDAR echo signals become significantly complex due to various interference factors. Therefore, it is necessary to validate the performance of the proposed method on real echo signals. We tested using a LiDAR prototype independently developed by our laboratory, employing the SPLPL90-3 laser transmitter produced by OSRAM and an avalanche photodiode (APD) receiver from Hamamatsu China. The laser wavelength is 905 nm, the pulse width is 20 ns, and the response frequency is 230 MHz. Detailed experimental parameters of the LiDAR system are listed in Table 5. The key parameters of the VMD-CPO-IWT algorithm are shown in Table 6. The distance l between the LiDAR and planar target was set to 10 m and 25 m, respectively, with a tilted wooden target of 0.3 reflectivity. The experimental setup is shown in Figure 13. The parameters in this experiment are designed to simulate the practical challenges LiDAR faces when detecting dark targets at medium distances. The objective is to rigorously evaluate the robustness of the proposed algorithm under low *SNR*_in_ conditions in a controlled environment.

**Figure 14:**
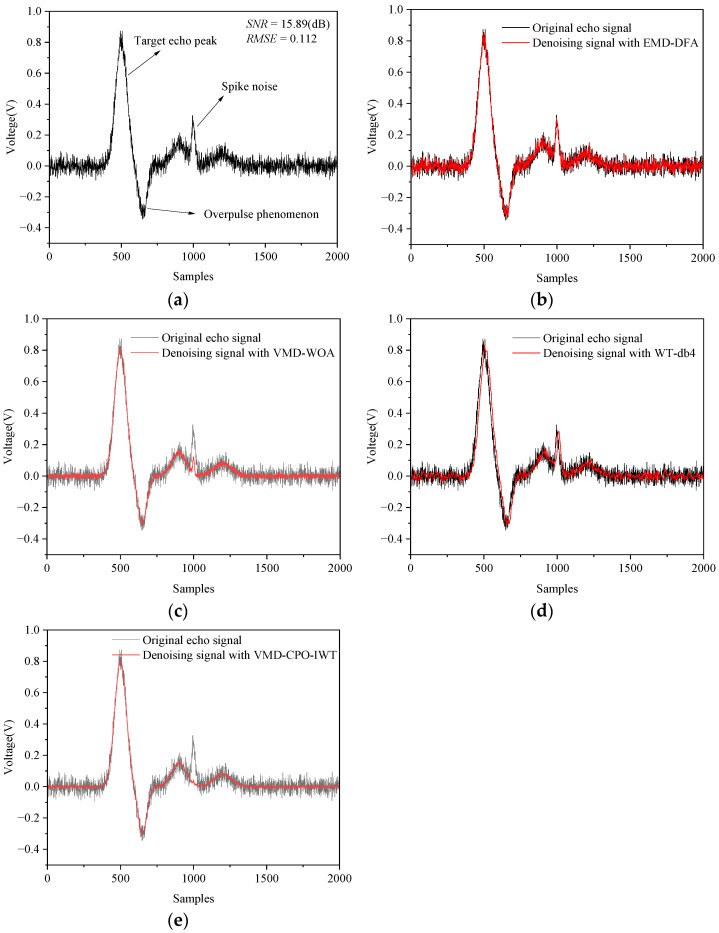
Comparison of denoising results for LiDAR echo signals (l=10 m): (**a**) Original echo signal; (**b**) EMD-DFA method; (**c**) VMD-WOA method; (**d**) WT-db4 method; (**e**) VMD-CPO-IWT method.

**Figure 15:**
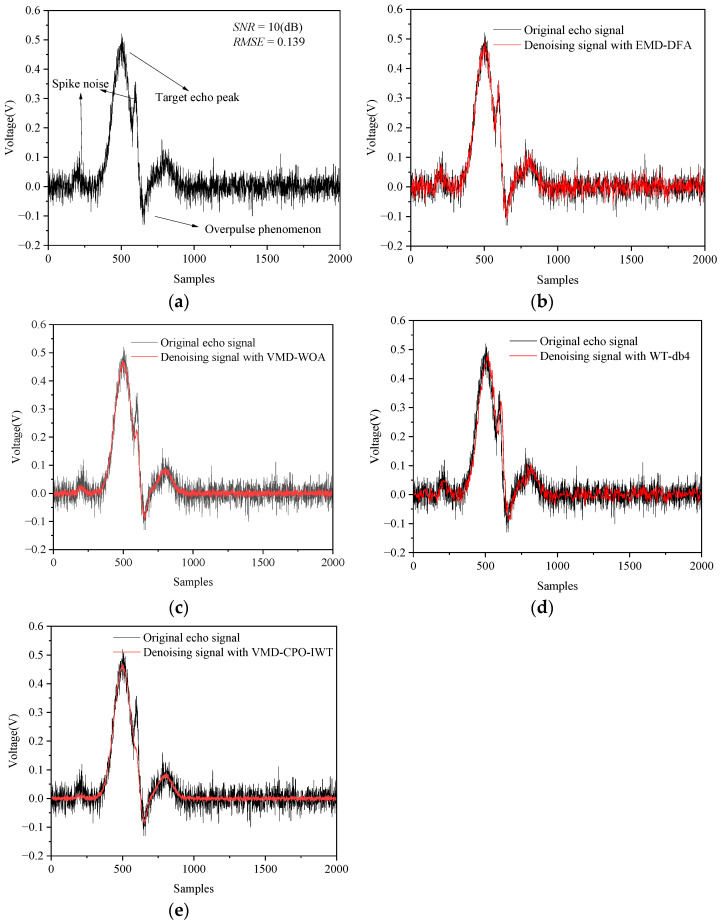
Comparison of denoising results for LiDAR echo signals (l=25 m): (**a**) Original echo signal; (**b**) EMD-DFA method; (**c**) VMD-WOA method; (**d**) WT-db4 method; (**e**) VMD-CPO-IWT method.

The measured echo waveforms acquired by the oscilloscope are shown in Figure 14 and Figure 15. It can be observed that the amplitudes of the two echo signals differ significantly at different measurement distances. When l=10 m and l=25 m the peak values of the LiDAR echo signals are 0.8 V and 0.46 V, respectively. This occurs because the intensity of LiDAR echo signals is inversely proportional to the square of the distance. Furthermore, the results indicate that the echo signals contain not only Gaussian white noise but also high-frequency spike pulse noise generated by rapid switching of circuits in specific sampling intervals.

As shown in Figure 14, signals processed by WT (db4) and VMD-WOA still exhibit numerous “burrs” and considerable fluctuations. EMD-DFA demonstrates the poorest denoising effect, with substantial Gaussian white noise remaining in the processed signal. Additionally, these three methods perform inadequately in suppressing high-frequency spike pulse noise. In contrast, the proposed VMD-CPO-IWT algorithm effectively eliminates Gaussian white noise throughout the signal, smoothes the waveform, and suppresses spike pulse noise. Table 7 presents denoising performance evaluations of different algorithms at l=10 m. Compared to the other three methods, the proposed algorithm achieves the highest *SNR* (31.21 dB) and smallest *RMSE* (0.042) for the denoised signal.

Figure 15 presents denoising test results for LiDAR echo signals under low *SNR* conditions. It can be observed that the VMD-CPO-IWT algorithm still achieves optimal denoising performance, effectively filtering out Gaussian white noise, while the other three methods yield unsatisfactory results. Table 8 lists denoising evaluations of various algorithms at l=25 m. The VMD-CPO-IWT algorithm attains an *SNR_out_* of 23.64 dB, higher than the other three methods, and simultaneously achieves the smallest *RMSE* of 0.052. These experimental results demonstrate that the proposed VMD-CPO-IWT method exhibits strong denoising capability for long-distance, low-*SNR* LiDAR echo signals. This superiority is attributed to: Adaptive parameter selection ensuring minimal energy loss during signal decomposition; WD effectively separating noise-dominant modes from pure signal components; IWT further filtering Gaussian white noise and smoothing the signal.

## 5. Discussion

While this study has comprehensively demonstrated the efficacy of the proposed CPO-VMD-IWT algorithm for denoising single-point LiDAR echo signals, its foundational framework, rooted in adaptive signal decomposition and noise separation, opens up several promising avenues for future research. The potential extensions of this work are primarily in three directions:

Firstly, the exploration of cross-disciplinary applications. The core strength of the CPO-VMD-IWT method lies in its generalizability for processing nonlinear and non-stationary signals. This suggests its potential applicability beyond LiDAR, particularly in domains like biomedical engineering (e.g., for denoising electrocardiogram (ECG) or electroencephalogram (EEG) signals to isolate physiological artifacts) and geophysics (e.g., for enhancing seismic signals by suppressing ground-roll noise). Future work will involve validating the algorithm’s performance and potentially tailoring it to the specific characteristics of these diverse signal types.

Secondly, the adaptation for advanced 3D sensing systems. Modern Flash and scanning LiDAR systems generate massive datasets comprising thousands of independent echo signals from different pixels or points. The proposed method, which operates on a per-signal basis, is inherently suitable for such architectures. The primary challenge shifts from algorithmic principle to computational efficiency. A key future direction is the development of a high-throughput, parallel computing implementation (e.g., leveraging GPU acceleration) to enable real-time denoising across all channels, thereby significantly enhancing the quality and reliability of the entire 3D point cloud under challenging detection conditions.

Finally, a systematic investigation into the algorithm’s robustness under extreme environmental conditions. Although the method shows theoretical promise in handling complex disturbances, its performance under practical scenarios with significant atmospheric perturbations, such as fog, rain, or dust, requires rigorous empirical validation. Future work will include controlled laboratory experiments and extensive field tests across various weather conditions to quantitatively assess the algorithm’s limitations and strengths, ensuring its practicality for real-world applications in autonomous navigation and remote sensing.

Through these investigations, the CPO-VMD-IWT algorithm can evolve from a specialized solution for LiDAR denoising into a versatile and robust signal-processing platform with broad impact across multiple scientific and engineering disciplines.

## 6. Conclusions

This paper proposes a novel joint denoising algorithm named VMD-CPO-IWT for LiDAR echo signals. The method first employs CPO to optimize VMD parameters, including the number of modes K and the quadratic penalty factor α. Subsequently, the input signal is decomposed using the optimized VMD parameter combination, and relevant modes are selected for signal reconstruction based on the WD between decomposed modes and the original signal. Following these steps, an improved wavelet denoising method is further applied to the reconstructed signal to achieve enhanced performance. The practicality and accuracy of the proposed method were validated through denoising experiments on multiple simulated and real signals under various conditions. By denoising four typical nonlinear non-stationary signals (Bumps, Blocks, Heavysine, and Doppler), the performance of VMD-CPO-IWT was compared with several other algorithms. Results demonstrate that VMD-CPO-IWT outperforms others in both *SNR_out_* and *RMSE*. Particularly for the Doppler signal with *SNR_in_* = −5 dB, the processed *SNR_out_* reaches 10.3 dB, representing a 15.3 dB improvement, with an *RMSE* of only 0.118. Finally, comparative denoising experiments were conducted on echo signals using our self-developed LiDAR prototype. Experimental results prove that the proposed method effectively suppresses Gaussian white noise and high-frequency spike pulse noise, preserves useful signals, and enhances the quality of LiDAR echo signals. This approach can be applied to weak LiDAR echo signal detection, significantly improving subsequent threshold timing discrimination accuracy.

## Figures and Tables

**Table 1:** Denoising performance of different methods for the noisy Bumps Signal (*SNR_in_* = 4 dB).

Method	WT-db4	VMD-WOA	EMD-DFA	VMD-CPO-IWT
*SNR_out_* (dB)	13.51	14.74	10.03	17.25
*RMSE*	0.3501	0.2925	0.5419	0.1736

**Table 2:** Average execution time comparison of different denoising methods.

Method	WT-db4	VMD-WOA	EMD-DFA	VMD-CPO-IWT
Average execution time (s)	0.51 ± 0.08	10.5 ± 2.1	0.82 ± 0.15	12.2 ± 2.9

**Table 3:** Denoising performance of three methods under CPO, WOA, and PSO optimized parameters.

Methods	CPO-Based Method	WOA-Based Method	PSO-Based Method
*SNR_out_* (dB)	15.792	14.680	14.791
*RMSE*	0.2987	0.3426	0.3279

**Table 4:** *RMSE* of each threshold method under different *SNR_in_*.

*SNR_in_* (dB)	*N* (ns)	VisuShrink	Rigsure	ISURE
−5	100	18.932 00	15.38900	15.09200
	75	27.10900	21.40900	20.45200
	60	151.80000	40.73200	25.58900
	50	164.83000	81.40100	29.05200
0	100	6.75010	4.93210	4.67200
	75	9.70560	6.72010	6.28970
	60	138.36000	10.27000	8.07930
	50	150.26000	47.87900	9.28350
10	100	2.00320	1.43290	1.46100
	75	3.34950	2.15900	1.87020
	60	131.43000	3.11960	2.30860
	50	142.37000	10.17000	2.78930
15	100	0.46913	0.45731	0.48251
	75	0.83592	0.61901	0.62538
	60	128.89000	0.94187	0.78942
	50	138.13011	2.35920	0.89550

**Table 5:** Experimental parameters of the LiDAR system.

System Parameter	Value
Detector responsivity (A/W)	70
Receiver diameter (mm)	20
Laser average transmitted power (W)	15
Pulse width (ns)	20
Atmospheric transmittance	0.979
System optical transmission efficiency	0.992
Laser wavelength (nm)	905
Sampling rate (GHz)	1
Beam divergence angle (mrad)	1
Trigger repetition rate (KHz)	200

**Table 6:** Key parameter settings for the CPO-VMD-IWT algorithm.

Module	Parameter	Value
CPO	Population size	30
	Maximum lteration	50
	Search bounds for K	[2, 15]
	Search bounds for α	[200, 10,000]
IWT	Wavelet basis	sym4
	Decomposition level	3
	Thresholding function	Soft thresholding
	Threshold selection method	ISURE

**Table 7:** Denoising evaluation of different algorithms for LiDAR echo signals at (l=10 m).

Methods	EMD-DFA	VMD-WOA	WT-db4	Ours
*SNR_out_* (dB)	23.74	28.92	25.42	31.21
*RMSE*	0.083	0.056	0.069	0.042

**Table 8:** Denoising evaluation of different algorithms for LiDAR echo signals at (l=25 m).

Methods	EMD-DFA	VMD-WOA	WT-db4	Ours
*SNR_out_* (dB)	14.03	20.73	16.72	23.64
*RMSE*	0.108	0.077	0.082	0.052

## Data Availability

Data underlying the results presented in this paper can be obtained from the authors upon reasonable request.
